# Barriers and facilitators for the implementation of Tuina (Jingjin) for neck pain in primary care settings in China: a qualitative study protocol

**DOI:** 10.3389/fmed.2026.1778187

**Published:** 2026-04-01

**Authors:** Zhichao Gong, Yinyan Gao, Xiaoyu Luo, Jiangshan Li, Wu Li, Xinyin Wu

**Affiliations:** 1 Department of Acupuncture, Massage and Rehabilitation, The Second Affiliated Hospital of Hunan University of Chinese Medicine, Changsha, Hunan, China; 2 College of Acupuncture, Massage and Rehabilitation, Hunan University of Chinese Medicine, Changsha, Hunan, China; 3 Department of Epidemiology and Health Statistics, Xiangya School of Public Health, Central South University, Changsha, Hunan, China; 4 Department of General Medicine, Xiangya Hospital, Central South University, Changsha, Hunan, China

**Keywords:** CFIR, implementation science, Jingjin Tuina, neck pain, primary health care, qualitative research

## Abstract

**Introduction:**

Neck pain is the fourth leading cause of disability worldwide. In China, primary care institutions face considerable challenges in the management of neck pain, primarily due to insufficient allocation of medical resources, resulting in an overreliance on pharmacological interventions in clinical practice. As an evidence-based and standardized non-pharmacological therapy, Tuina (Jingjin) offers distinct advantages in primary care settings, including high safety profiles and strong patient acceptance. However, its application and dissemination in frontline primary healthcare practice remain insufficient. The primary aim of this study will be to identify the barriers and facilitators for implementing Tuina (Jingjin) in primary care settings in China from the perspectives of multiple stakeholders.

**Methods:**

This qualitative study will utilize the five core domains of the Consolidated Framework for Implementation Research (CFIR 2.0)–Intervention Characteristics, Outer Setting, Inner Setting, Individual Characteristics, and Process–to inform the development of a semi-structured interview guide and data analysis. Recruitment will target patients with neck pain, traditional Chinese medicine general practitioners, and institutional managers from geographically diverse primary care institutions across China, with the sample size contingent upon achieving information saturation.

**Discussion:**

This study will contribute to the understanding of the multi-level factors that influence the implementation of Jingjin Tuina and provide strategies to enhance the adoption of this evidence-based intervention in primary care settings with limited resources.

## 1 Introduction

Neck pain is a global health problem recognized as the fourth leading cause of disability worldwide (1). The disease burden continues to escalate in parallel with demographic aging and the increasing prevalence of sedentary lifestyles. Epidemiological evidence demonstrates a marked upward trend in neck pain-related primary care consultations, underscoring its public health significance as a highly prevalent and frequently occurring condition (2). Currently, an overreliance on analgesic medications is observed in primary care settings (3, 4). Clinical evidence indicates that the misuse of these drugs can lead to serious adverse reactions, including gastrointestinal bleeding and an increased risk of mortality (5, 6), revealing critical safety concerns within prevailing treatment paradigms.

Tuina, a traditional Chinese medicine (TCM) technique, has been formally recommended in clinical guidelines as a first-line, non-pharmacological intervention for neck pain (7, 8). This study focuses specifically on Jingjin Tuina, which employs distal acupoint selection based on the Jingjin theory of TCM to effectively manage neck pain–both its clinical effect (9) and mechanistic foundations (10, 11) have received empirical validation. The distal application characteristic of this technique avoids direct cervical stimulation while simultaneously reducing treatment discomfort and enhancing patient tolerance. Crucially, compared to local manipulation approaches, Jingjin Tuina combines greater safety (eliminating requirements for pre-intervention imaging) with enhanced primary care applicability (featuring operational accessibility without necessitating specialized certification), rendering it optimally aligned with both conventional training frameworks and clinical demands within resource-constrained primary healthcare settings.

China’s ongoing initiative to integrate TCM into primary healthcare designates grassroots medical institutions as pivotal implementation centers. Paradoxically, Jingjin Tuina remains underutilized in these settings, despite its unique advantages for resource-constrained environments. This implementation gap substantially compromises effective neck pain management at the primary care level, generating an urgent need to identify context-specific implementation barriers.

We aim to investigate the barriers and facilitators to Jingjin Tuina adoption in Chinese primary healthcare settings, employing a qualitative approach guided by the Consolidated Framework for Implementation Research 2.0 (CFIR 2.0). The elucidation of these multidimensional determinants is foundational to developing precision implementation strategies that enhance the uptake and sustainability of this evidence-based intervention in real-world practice.

## 2 Methods

This qualitative study will be reported in accordance with the Consolidated Criteria for Reporting Qualitative Research (COREQ) guidelines to ensure comprehensive and transparent reporting.

### 2.1 Study design

In this qualitative study, we will recruit key stakeholders, including patients with neck pain, general practitioners of TCM at the primary level, and managers of primary medical institutions, for semi-structured interviews. The semi-structured interview guide (Supplementary File 1) was developed based on the CFIR 2.0 framework and refined through iterative consultations with clinical massage therapists and implementation scientists. The interview guide was pilot-tested by three experts, including TCM and/or evidence-based medicine specialists with extensive experience in treating neck pain.

### 2.2 Clinical innovation description

Jingjin Tuina is a standardized intervention based on the meridian-sinew theory of TCM. The technique targets specific sinew nodes located around the elbow, knee, wrist, and ankle joints–as documented in classical texts–rather than directly stimulating the cervical region (12, 13). This approach minimizes the risk of aggravating neck pain and enhances procedural safety, making it particularly suitable for primary care settings. The method primarily involves standardized pressing and plucking techniques, applied to defined acupoints, thereby avoiding the discomfort associated with spinal manipulation and reducing the occurrence of adverse events while improving treatment fidelity (Table 1).

**TABLE 1:** Intervention protocol of sinew channel Tuina.

Anatomical site of intervention	Selected acupoints	Duration per session (min)	Rationale and proposed mechanism of action
Upper limbs	Quchi (LI11)	2	Stimulate Jingjin (meridian sinew system in TCM) in the coronal plane of the neck to enhance lateral flexion and rotation.
	Tianjing (TE10)	2	
	Xiaohai (SI8)	2	
Lower limbs	Kunlun (BL60)	2	Stimulate the sagittal-plane and deep-layer Jingjin (meridian sinew system in TCM) to improve cervical flexion and extension.
	Taixi (KI3)	2	
	Xiangu (ST43)	2	
	Qiuxu (GB40)	2	

TCM, traditional Chinese medicine.

### 2.3 Study place and population

Recruitment will commence in May 2026 and continue for 6 months. We will purposively sample primary care practitioners of TCM involved in the management of neck pain, along with administrators from primary healthcare institutions (such as pain clinics or specialized services), treating patients with either acute conditions (including but not limited to fibromyalgia syndrome) (14) or chronic neck pain (e.g., osteoarthritis) (15). Recruitment will occur through non-institutional channels, including targeted advertisements distributed via professional associations (Chinese External Therapy Association, Tuina Society), social media platforms, and snowball sampling leveraging existing TCM clinical networks. Potential participants will be asked to contact the principal investigator via email, WeChat, or telephone to receive electronic participant information sheets and consent forms. Written consent will be confirmed through returned signed documents. Upon consent verification, interview scheduling will be coordinated directly with participants.

### 2.4 Sampling

Predefining sample sizes in qualitative research remains methodologically contentious. The core critique holds that specifying participant numbers *a priori* is logically untenable when critical themes remain emergent, offering limited potential to advance substantive understanding (16). To address this, our study will adopt thematic saturation as the termination criterion (17), ceasing recruitment when sequential data collection yields no novel insights.

Methodologically, the sample size will not be fixed *a priori* but will be guided by the principle of information power and thematic saturation. We will recruit participants until no new insights emerge from subsequent interviews. Given the heterogeneity of our sample (patients, TCM practitioners, and managers), we will monitor saturation within each stakeholder group separately. We anticipate that approximately 20–25 participants will be needed, however, recruitment will continue until each group reaches saturation. This approach aligns with Malterud’s framework (18), which advocates iterative inductive analysis and focused hermeneutic engagement to optimize saturation efficiency (19).

These methodological choices are fundamentally aligned with our research objectives: to rigorously examine clinical decision-making processes among primary care TCM practitioners managing neck pain, identify determinants influencing Jingjin Tuina application, and articulate practice implications derived from these insights.

### 2.5 Semi-structured interviews

This study will develop a semi-structured interview guide based on the CFIR-2.0. The interview protocol will be designed with open-ended questions that systematically address the five core domains of the CFIR framework: Innovation Characteristics, Outer Setting, Inner Setting, Individuals and process. Each domain includes multiple specific constructs, along with corresponding follow-up questions, to comprehensively identify facilitators and barriers to the implementation of Jingjin Tuina.

The interview guide will be developed through the structured consultation with TCM manual therapy experts. The final guide (Supplementary Material 1) will incorporate core questions addressing five CFIR-2.0 domains, supplemented by context-specific exploratory questions tailored to different stakeholder perspectives.

To mitigate professional allegiance bias, interviews will be conducted by GZC (a Tuina specialist) in the presence of either GYY (public health researcher) or LXY (general medicine researcher), who will take notes and ask follow-up questions from a different disciplinary perspective. The team will hold regular debriefing sessions to reflect on how the interviewer’s background may influence data collection. Each interviewer will maintain a reflexive diary to document preconceptions, emotional responses, and potential influences on the interview process. These reflections will be incorporated into team discussions during analysis to enhance interpretive awareness.

Each interview will last 40–50 min and follow a standardized protocol: participants will first receive detailed study information and interview guidelines, with an emphasis on their right to decline to answer any uncomfortable questions to protect their emotional well-being. After obtaining both verbal and written informed consent, the research team will audio-record the entire session. Immediate post-interview debriefings will be conducted to discuss key points and potential issues.

Audio recordings will undergo professional transcription, with all identifiable information will be redacted. To ensure transcription fidelity, a three-phase verification protocol will be implemented: (1) initial transcription by certified professionals; (2) researcher-led verification of audio-text alignment; and (3) clarification of sensitive content through respondent follow-up. All procedures will strictly comply with research ethics requirements, ensuring data integrity while safeguarding participant rights.

### 2.6 Analysis

Our analytical approach will combine framework analysis with inductive thematic analysis. After dual coding using NVivo 11, we will (1) conduct within-case analysis to identify individual implementation narratives, (2) perform cross-case comparisons between implemented and unimplemented sites, and (3) apply matrix analysis to examine interactions among CFIR domains. Emerging themes will be validated through member checking with key participants. Member checking will be conducted with a subset of key participants (approximately 5–7, representing all stakeholder groups) to enhance the credibility of our findings. Each participant will receive a one-page summary of emerging themes and illustrative quotes relevant to their stakeholder perspective. They will be invited to comment on whether the interpretations resonate with their experiences and to offer additional insights. Feedback will be used to refine themes and ensure that our analysis reflects the participants’ perspectives. The aim is dialogic validation rather than simple verification; we will engage in discussion with participants to co-interpret findings where appropriate.

For cross-case comparisons, we will categorize primary care sites as “implemented” if they have offered Jingjin Tuina as a routine service for neck pain management for at least 6 months prior to data collection, and as “unimplemented” if they have not adopted Jingjin Tuina or have only piloted it occasionally. This categorization will be based on the information provided by managers and practitioners during interviews.

The research team will implement a rigorous four-phase analytical procedure:

#### 2.6.1 Phase 1: data familiarization and preliminary coding

The research team will conduct an extensive data familiarization process, during which all members will systematically review the interview transcripts to ensure a comprehensive understanding. Initial coding will be performed independently by two coders (ZDJ and JZL) using the first two interview transcripts. A deductive approach will be used to align text segments with specific CFIR 2.0 framework constructs, while open coding will be applied to capture ambiguous or unclassifiable text. Through iterative team discussions, the researchers will consolidate the coding results, clearly define each construct within the study context, and develop a preliminary coding manual.

#### 2.6.2 Phase 2: systematic thematic coding and framework refinement

Coders will independently apply the coding manual to the remaining transcripts, ensuring consistency in the application of constructs. After the coding each transcript, the research team will conduct alignment discussions to integrate findings and produce a consolidated memorandum of CFIR constructs. The coding manual will be continuously refined to incorporate newly identified constructs to maintain analytical comprehensiveness.

#### 2.6.3 Phase 3: software-assisted analysis and construct development

NVivo V.11 qualitative analysis software (QSR International, Melbourne, Australia) will be used to enhance analytical rigor. The systematic search function of the software ensures no relevant data segments are overlooked, with findings integrated into the summary memorandum. For text segments not captured by existing constructs, the team will inductively develop new constructs, thereby preserving analytical flexibility and innovation. Coded data will be mapped to CFIR-2.0 constructs using matrix analysis, visualizing interactions between domains with concept maps.

#### 2.6.4 Phase 4: expert consensus and final analysis

A multidisciplinary expert workshop, including Tuina therapy practitioners, will be convened to systematically review the summary memorandum and identify the core facilitators and barriers to Jingjin Tuina implementation. Investigators WXY and LJS will oversee the analytical process to ensure methodological rigor and reliability. This multi-stage, multi-perspective approach will enable a comprehensive exploration of the key factors and underlying mechanisms shaping the implementation outcomes of Tuina therapy.

Consolidated Framework for Implementation Research will be employed as a flexible heuristic framework to sensitize our inquiry, and not as a fixed coding dictionary. While deductive coding will map data to CFIR constructs, we intend to remain attentive to text segments that do not fit existing constructs, allowing for inductive development of new themes and modifications to the framework as needed.

To ensure interpretive consistency, the two coders will meet after each transcript to discuss discrepancies and reach consensus through dialogue. A third researcher (GZC) will facilitate resolution of any persistent disagreements. This iterative consensus process prioritizes interpretive rigor over statistical agreement.

### 2.7 Study period

This qualitative research study will begin in March 2026. Key informants will be interviewed in the initial phase, with data collection completed by May 2026. Participant recruitment and in-depth interviews will continue through October 2026 until theoretical saturation is reached.

Data analysis will be performed from June to September 2026, with data coded using a constant comparative method within the CFIR 2.0 framework. The final analysis will be completed by October 30, 2026. After the final phase of the study, manuscript preparation and evidence-based strategy development will be concluded on November 30, 2026.

### 2.8 Patient and public involvement

The patients and the public were not involved in the design of this protocol.

### 2.9 Ethics and dissemination

This study involves human participants (patients with neck pain, TCM practitioners, and primary care managers) and has received ethical approval from the Ethics Committee of the Second Affiliated Hospital of Hunan University of Chinese Medicine (Approval No. 2025-KY-026-01). The study will be conducted in accordance with the Declaration of Helsinki and all applicable ethical guidelines.

To protect participants’ rights, we will implement comprehensive privacy safeguards: all interview transcripts will be anonymized by removing personally identifiable information and assigning unique codes (e.g., R1, R2). The research team will conduct dual verification of transcripts to ensure confidentiality.

Prior to enrollment, researchers will thoroughly explain the study’s purpose, potential risks, and privacy protection measures, with particular attention to the vulnerable status of general practitioners in this context. Participation is entirely voluntary and requires both oral and written informed consent. Participants may withdraw at any time without providing justification.

### 2.10 Data management

All research data will be managed according to the Findable, Accessible, Interoperable, and Reusable (FAIR) principles. Electronic data will be stored on password-protected institutional servers with regular backup. Physical documents will be kept in locked cabinets in the investigator’s office. All data will be retained for 5 years after study completion, in accordance with institutional policy.

### 2.11 Dissemination of study findings

Our report will comply with the standards for reporting qualitative research. We will share the results of this study through publications in peer-reviewed journals, as well as presentations and panel discussions involving academics, policymakers, and clinicians at scientific conferences.

## 3 Discussion

This pioneering study represents the first systematic investigation into the clinical reasoning processes employed by TCM general practitioners at the grassroots level when prescribing Jingjin Tuina for neck pain management. Given the critical evidence gap regarding acupoint selection and treatment protocols in primary care settings, our research addresses an urgent need by elucidating how practitioners develop and implement Jingjin Tuina. To ensure both scientific rigor and practical relevance, the study design incorporates stakeholder engagement through a dedicated steering committee of TCM clinicians and implementation experts, enabling a comprehensive examination of clinical decision-making patterns.

Our collaborative approach specifically explores how grassroots practitioners prioritize Tuina modalities within treatment pathways while simultaneously mapping the complex constellation of factors influencing their clinical reasoning, including patient presentation metrics, therapeutic goals, evidence accessibility, and practitioner expertise. The investigation further captures practitioners’ concerns regarding treatment implementation barriers, thereby identifying critical knowledge gaps requiring resolution. Understanding these reasoning mechanisms will generate transformative insights into optimizing the clinical application of Jingjin Tuina, directly informing the development of culturally responsive training programs that integrate practitioner experiences with theoretical frameworks.

The qualitative dataset generated in this study will serve as foundational evidence for clinical protocol refinement, offering substantial cross-disciplinary value. Beyond immediate practical applications for neck pain management, the findings will catalyze advancements in three key domains: informing future mixed-methods research designs, enhancing undergraduate TCM curriculum development, and guiding healthcare policy reform for integrative pain management. This comprehensive approach ultimately creates a ripple effect across healthcare education, clinical practice, and research methodology, addressing a significant yet underexplored dimension of evidence-based traditional medicine integration.

These solutions significantly improve intervention fidelity in primary care settings, simultaneously elevating care quality for patients with neck pain and establishing a transferable framework for integrating traditional medicine into contemporary healthcare systems. This protocol will generate in-depth qualitative data on multi-level determinants of Jingjin Tuina implementation in primary care. The insights gained will inform the development of context-sensitive strategies to enhance uptake and sustainability, and will provide a foundation for future mixed-methods research and policy recommendations.

This study acknowledges the following limitations: first, the restriction of participant recruitment to Hunan Province may limit the generalizability of our findings due to regional variations in the implementation of traditional Chinese medicine (TCM) policies across China. Nevertheless, rigorous adherence to guidelines for complex health intervention research ensures methodological robustness despite the sample size constraints inherent in qualitative designs. Through in-depth contextual analysis, these findings will provide critical insights for clinicians to (1) identify and rectify maladaptive cognitive beliefs in patients, (2) design individualized management plans, and (3) optimize clinician-patient communication strategies, ultimately advancing innovation in the application of Jingjin Tuina for neck pain management in primary care. Regarding potential biases, social desirability bias in interviews may lead participants to underreport barriers. To mitigate this, we will ensure confidentiality and use open-ended questions that normalize the discussion of challenges. Another pitfall is the risk of over-interpretation due to the lead interviewer’s clinical background; we will address this through reflexive practices and multi-disciplinary analysis. In terms of ethical considerations, to minimize any unintended burden on participants, we will allow for breaks during interviews and offer the option to withdraw at any time. If any distress is observed, we will provide appropriate support and referral services. To address these limitations, future research should conduct multi-site validations across diverse socioeconomic contexts and employ mixed-methods approaches to quantify the impacts of specific barriers (e.g., training gaps on implementation outcomes) while expanding sample sizes.

In conclusion, this study will examine the mechanisms influencing the implementation of Jingjin Tuina in primary care settings using the CFIR-2.0 framework, revealing dynamic interactions among multi-level factors. Although the findings are geographically limited to China, they offer methodological advancements for scientifically evaluating the implementation of traditional medical therapies. Future research should adopt mixed-methods designs to quantitatively assess the efficacy of identified implementation strategies and explore integrative models that combine Jingjin Tuina with other primary healthcare interventions. Such expansion would enhance generalizability and support evidence-based integration of traditional therapies into mainstream healthcare systems.
