# Supplementary material for: Barriers and facilitators for the implementation of Tuina (Jingjin) for neck pain in primary care settings in China: a qualitative study protocol
Source: Front Med (Lausanne). 2026 Apr 1;13:1778187. doi: 10.3389/fmed.2026.1778187 (PMC13078968; doi:10.3389/fmed.2026.1778187)
Supplement: Supplementary file 1 [file Supplementary_file_1.docx]

**Identifying Barriers and Facilitators to the Implementation of Jingjin Tuina for Neck Pain in Primary Care: A Qualitative Interview Guide (Patient Version)**

**Opening Statement​**​

I am a researcher from the Department of Tuina, the Second Hospital of Hunan University of Chinese Medicine. Thank you for participating in this study. We are investigating the implementation of Jingjin Tuina for neck pain in primary care settings. Our objective is to identify the barriers and facilitators to its delivery and to understand the format in which patients prefer to receive this intervention.

**Technical Context: Jingjin Tuina** is a standardized manual therapy guided by Jingjin (meridian sinew) theory, involving the stimulation of specific distal acupoints on the limbs to achieve therapeutic efficacy.

**Confidentiality & Consent:** This interview will take approximately 30 minutes. There are no standard answers; we encourage you to share your authentic experiences. The conversation will be audio-recorded for analysis, and we guarantee strict confidentiality. With your consent, we will formally begin. We appreciate your support for our research.

**Basic Information​**​

**Your age** ______ year (of age) ；**sex:** □ male □ female

**occupation**： □ Government Agencies/Institutions □ Enterprise employees □ self-employed □ peasant □ retire □ student □ Unemployed □ other______

**What is your highest education level?** □ Primary school and below □ junior middle school, junior high school □ High school/vocational school □ junior college □ Bachelor's degree or above

**Which area do you live in?** □ urban area □ small towns □ rural area

**Duration of neck pain**：□ < 3-month acute；□ 3 months-1 year (subacute)；□ > 1 year (Chronic: According to international standards, pain that persists or recurs for more than 3 months is typically defined as chronic pain. In this study, we define chronic pain as lasting more than 1 year to more than clearly distinguish patients with long-term suffering.)

**Have you tried other treatments for neck pain before this visit? (Multiple selections allowed)**

□ Medicated patches；□ Massage parlor/health spa massage；□ Oral analgesics/muscle relaxants

□ Physical therapy (e.g., acupuncture, traction, electrotherapy, etc.)；

□ Hospital visits (Orthopedics, Rehabilitation, Pain Management, etc.)

□ Private Traditional Chinese Medicine Clinic/Tui Na (Massage) Studio；

□ self-exercise/activity；□ other：_________

**How many times have you visited this community health center/township health center for neck pain?** □ The first time □ 2-3 times □ 4 or more times

**What are your primary methods of medical expense payment? (Multiple selections allowed)**

□ urban employee medical insurance □ Urban and Rural Residents' Medical Insurance

□ New Rural Cooperative Medical Scheme □commercial insurance

□at one's own expense □ other______

**What are the primary reasons for choosing to seek medical care at community health centers/township health centers? (Multiple selections allowed)**

□ Close to home/convenient □ Low cost/high reimbursement rate □ Recommendations from friends or family □ Trust the doctors here □ Convenient registration with short waiting time

□ Overcrowded large hospitals/Difficulty in registration □ other______

**Interview Outline**

**Intervention Characteristics**

1. **Cognitive Study on Jingjin Tuina Therapy for Cervical Pain（Evidence Strength & Quality）**
2. How do you perceive the therapeutic effects of the Jingjin Tuina therapy for cervical pain? (e.g., treatment efficacy, diagnostic and therapeutic procedures, level of comfort)
3. **Relative Advantage**
4. The physician primarily applied acupressure to the acupoints on your arm or leg to treat cervical pain (Jing-Jin Tuina).
5. Do you think this differs from the traditional neck massage you previously performed?
6. How does your task differ from **other non-massage interventions** (including acupuncture, oral medications, rehabilitation devices, etc.)?

**For instance, advantages** (e.g., relative safety, relative comfort, etc.); and **disadvantages**, such as questions or areas of discomfort (e.g., why does pressing the limbs treat the neck? Is the effect perceived to be slower?); do you think this method is suitable for patients with cervical pain like yourself? Why?

1. **Adaptability**
2. You have just experienced Jingjin Tuina here. Do you think it should be promoted at the grassroots level?For any necessary adjustments (e.g., reducing pressure, adding acupoints), please provide detailed explanations (including: technical training for physicians, additional equipment preparation, improved environmental conditions, enhanced physician-patient communication, and increased health education lectures on preventive measures).
3. **Complexity**
4. If the acupoints need to be adjusted based on the location of your pain, would this affect your confidence in the treatment? How do you perceive the operational difficulty of this therapy?

**Outer Settings**

1. **Local Perspective**
2. Does your community trust massage therapy? Are there prejudices such as 'massage is only for health maintenance (e.g.,' unscientific 'or' fear of damage ')'?
3. Do your family members support your choice of massage therapy over Western medicine?
4. **Local Conditions**
5. Does the convenience of transportation in this area affect your access to community healthcare? Does the network coverage support remote appointment scheduling?
6. **External Policy and Incentive**
7. Are there any relevant medical insurance policies or Traditional Chinese Medicine (TCM) policies that may assist in your decision to undergo massage therapy?

**Inner Settings**

1. **Organizational Structure Characteristics**
2. Does the community center's grassroots orientation raise concerns about the quality of medical services?
3. **Collaboration and Communication**
4. Does the physician collaborate with rehabilitation or Western medicine specialists to develop the treatment plan? Would you prefer interdisciplinary services (e.g., massage + physiotherapy)?
5. **Organizational Culture and Implementation Atmosphere**
6. Do you believe the community encourages innovation? (e.g., promoting new therapies) Do physicians actively advocate for Jingjin Tuina?
7. **Available Resource**
8. Do you believe the resources of primary healthcare institutions can meet your needs (e.g., office workers)?

**Individuals Domain**

1. **Patient Knowledge and Beliefs**
2. Do you understand the principle of 'remote acupoint selection'? If No, does it undermine your confidence in treatment?
3. **Patient Characteristics**
4. Does your health literacy (e.g., acupoint awareness) influence treatment compliance? Would individuals with low pain tolerance refuse strong manual stimulation?

**Process Domain**

1. **Planning and Mobilization**
2. How far in advance would you like to schedule a massage session? Which method-brochures or short videos-would be more effective in attracting you to learn about the new therapy?
3. **Opinion Leaders and Their Supporters**
4. Does the hands-on experience with senior physicians provide greater reassurance? Can patient recovery sharing sessions encourage your participation?
5. **Implementation and Evaluation**
6. Does the physician inquire about your feedback (e.g., regarding force adjustment) during treatment? How would you like to participate in efficacy evaluation (e.g., by completing a scale)?
7. **Sustainability Strategy**
8. If a self-help manual for families (such as an acupoint diagram) is provided, could it enhance your willingness to adhere to the long-term regimen? If No, what are the key factors that improve your long-term adherence?

**Identifying Barriers and Facilitators to the Implementation of Jingjin Tuina for Neck Pain in Primary Care: A Qualitative Interview Guide (Practitioner and Administrator Version)**

**Opening Statement**​​

I am a researcher from the Department of Tuina, the Second Hospital of Hunan University of Chinese Medicine. Thank you for participating in this study. We are investigating the implementation of Jingjin Tuina for neck pain in primary care settings. Our objective is to identify the barriers and facilitators to its delivery and to understand the format in which patients prefer to receive this intervention.

**Technical Context: Jingjin Tuina** is a standardized manual therapy guided by Jingjin (meridian sinew) theory, involving the stimulation of specific distal acupoints on the limbs to achieve therapeutic efficacy.

**Confidentiality & Consent:** This interview will take approximately 30 minutes. There are no standard answers; we encourage you to share your authentic experiences. The conversation will be audio-recorded for analysis, and we guarantee strict confidentiality. With your consent, we will formally begin. We appreciate your support for our research.

**Basic Information**

Your age：_____

Your gender：_____

Your education level is：_____

Years of medical practice：_____

Management period：______

Your role：□Center Director □Deputy President of the Business Division □Head of Medical Services □Head of the Traditional Chinese Medicine Department

Your healthcare facility level (Community Health Service Center/Township Health Center, etc.)：_____

Has received massage therapy-related training? Duration and content of the training：_____

Proportion of Traditional Chinese Medicine services in your institution：

□<20% □20%-50% □>50%

Growth rate of investment in traditional Chinese medicine over the past three years：

□<5% □5%-10% □>10%

**Interview Outline**

**Intervention Characteristics**

1. **Cognitive Study on Jingjin Tuina Therapy for Cervical Pain（Evidence Strength & Quality）**
2. What is your level of familiarity with **Jingjin Tuina** (emphasis: based on meridian tendon theory, primarily involves acupressure at distal acupoints of the limbs to treat neck pain)? What proportion does it account for in your therapeutic approach to cervical pain? If No, what primary intervention method do you primarily use in your daily practice? (Local massage? Visceral massage? Acupuncture?)
3. Do you understand the clinical efficacy of massage (meridian and tendon massage) in treating neck pain(evidence-based)？

- If Yes: How did you get this information? To what extent do these pieces of evidence influence your views or practices?
- If No: Do you have evidence-based evidence of other interventions for neck pain?

1. **Relative Advantage**
2. Compared with other commonly used methods such as pharmacotherapy and physical therapy, what differences do you perceive in the therapeutic effects of Jingjin Tuina? Are you willing to use Jingjin Tuina at the grassroots level?

- If Yes: Why would you prefer to use Jingjin Tuina therapy at the primary care level, and what are the advantages of its application there (e.g., side effects, therapeutic characteristics, etc.)?
- If No: Why are you unwilling to use it? What are your main concerns (e.g., operational difficulty, target population, treatment duration, etc.)?

1. **Adaptability**
2. During the application of Jingjin (a traditional Chinese massage technique) at the primary care level, can modifications or adjustments (time, intervention site) be made to better facilitate its implementation in grassroots settings?
3. **Complexity**
4. In your daily clinical experience with cervical pain, what is the complexity of applying Jingjin Tuina techniques? (e.g., precision of syndrome differentiation and acupoint selection, control of manipulation force, efficacy evaluation)

**Outer Settings**

1. **Local Views (including Patient Needs & Resources）**
2. Among the patients with cervical pain you have treated, what is your perception of their general attitude toward Jingjin Tuina therapy? (e.g., trust and support, skepticism and concerns, preference for medication, etc.)
3. Have you encountered patients who refuse massage therapy due to cultural beliefs (e.g., "Massage only relieves tension and cannot cure diseases; they prefer injections and oral medications")? What are the three core demands of patients with cervical pain for massage therapy in daily clinical practice? (e.g., rapid pain relief, avoidance of medication, low cost) Could you provide examples and share your perspective?
4. **Cooperation and Contact**
5. Do you have technical collaborations with superior hospitals (such as our hospital) or other specialized institutions in the field of massage therapy? What are the forms of collaboration (e.g., remote consultations, expert guidance in rural areas, advanced training, case discussions)? To what extent do these collaborations meet your needs?
6. **External Policy and Regulation**
7. What are the impacts of current TCM-related policies (such as the '14th Five-Year Plan' for TCM development, which mandates the promotion of non-pharmacological TCM therapies) on the implementation of Jingjin Tuina therapy at the primary care level?
8. What is the coverage and reimbursement ratio of local medical insurance policies for Jingjin Tuina therapy? Do you believe this significantly impacts patients' choice of Tuina therapy? What specific issues exist in the reimbursement policy (e.g., item restrictions, excessively low reimbursement rates, cumbersome procedures)?
9. **Financial Support**
10. Are there any financial policies, projects, or funding initiatives to promote grassroots application of Jingjin Tuina?
11. **External Pressure**
12. Are there any general practitioners of Traditional Chinese Medicine (TCM) you know who use Jingjin Tuina techniques to treat neck pain? What are their perspectives on this matter? How have their viewpoints influenced your decision to utilize Jingjin Tuina? If surrounding private institutions offer “Tuina + package” services, would this compel you to enhance your service quality?
13. Does regular consultation with specialists from higher-level hospitals for cervical pain diagnosis and treatment weaken patients' willingness to choose your institution?

**Inner Settings**

1. **Structural Characteristics**
2. Does your institution provide Jingjin Tuina services? Are the necessary conditions for performing massage (such as venue, equipment, and personnel) available? If No, what do you consider to be the primary reason for the institution's failure to offer Jingjin Tuina services? How would you implement massage therapy (e.g., by referring patients to other institutions)?
3. **Networks & Communications**
4. What is the collaboration mechanism between you and the massage therapist in your institution (e.g., referral process, information communication, joint diagnosis and treatment)? Is the collaboration smooth? What are the main obstacles? Do you think Jingjin Tuina is suitable for implementation under this collaborative model?
5. **Urgency for Innovation**
6. Do you consider the promotion of Jingjin Tuina therapy for primary care patients with neck pain to be an urgent need for healthcare institutions? Why?
7. **Compatibility**
8. How well does the application of Jingjin Tuina align with your existing workflow in your environment?What are the potential problems or complications that may arise during this process?
9. **Relative Priority**
10. What is the priority of Jingjin Tuina of neck pain? Why?
11. **Organizational Incentives & Rewards**
12. Does your institution have incentive measures, performance rewards, or other mechanisms to promote the use of traditional Chinese medicine (TCM) techniques such as Jingjin Tuina (a type of acupressure therapy)? (Please provide specific details.) Has the institution incorporated the 'promotion of non-pharmacological therapies' into its annual objectives?

**7. Available Resources**

1. What resources currently exist to facilitate the implementation of Jingjin Tuina? (For example: specialized training/lectures for massage therapists, standardized operating manuals/process diagrams, joint outpatient/case discussions with the massage departments of superior hospitals, convenient referral platforms, patient education materials, etc.) Why?

**8. Access to Knowledge & Information**

1. Do you have any knowledge or information sources related to Jingjin Tuina? If so, please list them.

**Characteristics of Individuals**

1. **Capability**
2. Are you proficient in the knowledge and techniques of Jingjin Tuina therapy for cervical pain (such as Jingjin differentiation)? In which aspects are you more knowledgeable, and in which areas do you need further improvement?
3. As a manager, what do you consider to be the core values of Jingjin Tuina? (e.g., enhancing the distinctive features of Traditional Chinese Medicine and reducing the proportion of pharmaceuticals)
4. **Opportunity**
5. Have you received training support related to Jingjin Tuina? If available, through which channels did Jingjin acquire Tuina's knowledge and technology?
6. **Motivation**
7. Would you be willing to use Jingjin Tuina in the daily diagnosis and treatment of neck pain? Why? And under what circumstances would you prioritize Jngjin Tuina (e.g., when patients refuse Western medicine or have chronic pain)?
8. Have you experienced any cases with better or worse therapeutic outcomes Jingjin Tuina treatment for cervical pain in your daily clinical practice? Does this have any impact on your application of Jingjin Tuina?
9. If conditions permit, would you be willing to further study and promote Jingjin Tuina?

**Process Domain**

1. **Access to Knowledge**
2. The most effective training method in your opinion as:□Expert on-site mentoring □Standardized Video Library □WeChat Q&A Group
3. Minimum required training duration：□≤1 day □3 days □1 week
4. **Executing**
5. Should a massage efficacy tracking form be established? (e.g., follow-up rate and satisfaction rate for patients with neck pain); How to collect patient feedback? (e.g., QR code evaluation)
6. **Reflecting & Evaluating**
7. If a 'Patient Self-Service Massage Group' is initiated (e.g., under your guidance), would you be willing to undertake additional workload?
8. What kind of sustained support is expected from the superior hospital? □remote quality control □Consultation of Difficult Cases □Quota for advanced studies
